# The Fuzziness in Molecular, Supramolecular, and Systems Chemistry

**DOI:** 10.3390/molecules25163634

**Published:** 2020-08-10

**Authors:** Pier Luigi Gentili

**Affiliations:** Department of Chemistry, Biology, and Biotechnology, Università degli Studi di Perugia, Via elce di sotto 8, 06123 Perugia, Italy; pierluigi.gentili@unipg.it; Tel.: +39-075-585-5573

## 1. Introduction

The global challenges of the XXI century require a more in-depth analysis and investigation of complex systems [1]. A promising research line to better understand complex systems, and propose new algorithms and computing devices is natural computing. Natural computing is based on a fundamental rationale: every causal phenomenon can be conceived as a computation and every distinguishable physicochemical state of matter and energy can be used to encode information. Any physicochemical law can be exploited to make computations. For instance, quantum mechanics laws can be exploited to make quantum computing; the chemical kinetic laws can be used to make chemical computing; the laws of chaos to make chaos-computing, etc. On the other hand, we might draw inspiration from living beings with the exclusive attribute of using matter and energy to encode, collect, store, process, and send information [1,2]. Living beings show different information systems. Their basic information system is the cell, also called the biomolecular information system (BIS). In most multicellular organisms, we encounter nervous systems that constitute neural information systems (NISs). The defense systems that help repel antigens and disease-causing organisms are defined as immune information systems (IISs). Finally, most living beings live in societies, and the resulting aggregations constitute the so-called social information systems (SISs).

## 2. Artificial Intelligence and Fuzzy Logic

Among the natural information systems, particularly alluring for facing XXI century challenges, is the human nervous system (HNS). Its performances are astonishing. Based on a complex architecture of billions of nerve cells, our nervous system allows us to handle accurate and vague information by computing with numbers and words. Furthermore, it allows us to recognize variable patterns quite easily and make decisions in complex situations. Therefore, it is worthwhile trying to understand how it works and mimic it by developing artificial intelligence (AI). Within AI, fuzzy logic stands out as a good model of the human ability to compute with words and make decisions in complex circumstances [3,4]. Its descriptive and modeling power hinges on the structural and functional analogies it has with the HNS [5,6]. The entire architecture of the HNS is related to that of any fuzzy logic system. Our natural sensors play as fuzzifiers, our brain as a fuzzy inference engine, and our effectors as defuzzifiers. Every sensory system, physical and chemical, such as the visual or olfactory system, is constituted by a tissue of a spatially distributed array of sensory cells that behave as fuzzy sets [5,6]. Within each sensory cell, there is a multitude of sensory proteins that work as molecular fuzzy sets. The multiple information of any stimulus, i.e., its modality, intensity, spatial distribution, and time-evolution, is encoded hierarchically as degrees of membership to the molecular and cellular fuzzy sets. The imitation of these features allows us to design new artificial sensory systems with enhanced discriminative power due to different molecular fuzzy sets’ parallel activity. A concrete example is the recent implementation of biologically inspired photochromic fuzzy logic systems that extend human vision to the UV [7,8].

## 3. Neuromorphic Engineering and Chemical Artificial Intelligence

The mimicry of nonlinear neural dynamics is a promising alternative strategy to approach human intelligence performances. Surrogates of neurons can be achieved through either oscillatory or excitable or chaotic chemical systems in solution (i.e., wetware) [9,10] or the solid phase (i.e., hardware) [11,12,13]. In this Special Issue, Szaciłowski and his team present an experimental characterization of an optoelectronic device, constituted by a polycrystalline cadmium sulfide electrode [14]. Such a device realizes a type of short-term plasticity, i.e., the paired-pulsed facilitation (PPF). The PPF consists of an enhancement in the postsynaptic current when the excitatory signal frequency increases. This short-term memory effect confers to the device an appreciable power of recognizing hand-written numbers. Szaciłowski’s work blazes a trail for the optoelectronic implementation of neural network architectures that will allow the processing of fuzzy logic and recognition of variable patterns. Suffice to think that fuzzy logic has already been implemented through a pacemaker neuron model, such as the Belousov-Zhabotinsky reaction [15], and a chaotic neuron model, such as the “photochemical oscillator” [16,17]. When UV–visible radiation is chosen as a signal, it is straightforward to implement neuromodulation [18] and hence, fuzzy logic.

In the orthodox AI, fuzzy logic is processed through software running in digital electronic computers; it is even better if the electronic circuits are analog, since fuzzy logic is an infinite-valued logic. In the burgeoning field of chemical artificial intelligence (CAI) [19], unconventional chemical systems have been put forward for implementing fuzzy logic systems. Some examples can be found in the references [20,21,22,23,24,25,26]. The fundamental requirement is to have smooth analog input–output relationships between physicochemical variables, either linear or hyperbolic, but certainly not sigmoid. Sigmoid functions are adequate for processing discrete logics [27,28].

## 4. Cellular Fuzziness

The relentless investigation of the working principles of the cells or BISs has been revealing cellular fuzziness. Some proteins play within any cell as if they were the neurons of the “cellular nervous system” [6]. They are the protagonists of the signaling and genetic networks, and they make the cell capable of responding to ever-changing environmental conditions. As Fuxreiter points out in her perspective included in this Special Issue [29], often, a protein exists as a heterogeneous ensemble of conformers. For these proteins, the deterministic inference “amino-acidic sequence → 3D structure → function” is not applicable. In fact, the conformational ensemble may perform multiple functions, depending on the context. Such a collection of conformers looks like a macromolecular fuzzy set. The dynamical power of a protein to autonomously select a context-dependent function constitutes what we might name as its fuzzy inference engine. In their review, Jeffery and Liu tell us that there are moonlighting proteins in the metabolic network of a cell, in which one polypeptide chain performs more than one physiologically relevant biochemical or biophysical function [30]. The kind of function that is executed might depend on cellular localization, concentrations of substrates or ligands, or environmental stress. Any type of moonlighting protein is fuzzy because some of its copies can perform one function, some another, and some both functions simultaneously. As cellular conditions change due to metabolism and environmental conditions, the functions of these proteins change as well. Uversky informs us that within a cell, the supramolecular interactions between specific intrinsically disordered proteins and hybrid proteins, having ordered domains and intrinsically disordered protein regions, drive biological liquid–liquid phase transitions that form proteinaceous membrane-less organelles (PMLOs) [31]. PMLOs are intracellular hot spots that serve as organizers of cellular biochemistry. Such PMLOs are fuzzy, and their fuzziness resides in their compositional and compartmental variety and variability. Dodero and her team made the tangible experience of supramolecular fuzziness by investigating the interaction between a Transcription Factor and double-stranded DNA [32]. After annealing a proper DNA sequence and synthesizing a photosensitive surrogate of the GCN4 Transcription Factor, Dodero and her colleagues furnish experimental evidence of the protein-DNA complexation fuzziness by using different techniques, such as NMR, electrophoretic mobility shift assay, and circular dichroism spectroscopy. To monitor the conformational fuzziness of macromolecules and smaller molecules, Gentili relies upon the maximum entropy method to extract the distributions of conformers from any kinetic trace [6,33]. After determining the distribution, quantifying its fuzzy entropy is also possible [6,34].

## 5. Non-Arrhenius Kinetics

If we consider the conformational distributions of compounds, the original transition-state theory and the Arrhenius law might appear far-fetched. There is a peculiar distribution of conformers at every temperature, and every conformer traces its unique reactive path. It is not fair to define just one kinetic constant and one activation energy for all the coexistent conformers. It is necessary to add that both the original transition-state theory and the Arrhenius law have been already questioned by the most recent theoretical and experimental developments, as evidenced by Carvalho-Silva, Coutinho, and Aquilanti [35]. Quantum mechanical effects, such as tunneling and resonance, stochastic motions of particles in condensed environments, and non-equilibrium effects in classical and quantum formulations, are responsible for deviations from the traditional Arrhenius equation. In such situations, the transitivity function, defined in terms of the reciprocal of the apparent activation energy, measures the propensity for a reaction to proceed. The transitivity function provides a tool for implementing phenomenological kinetic models. In reference [36], Machado, Sanches-Neto, Coutinho, Mundim, Palazzetti, and Carvalho-Silva document the general scope of a transitivity code that can estimate the kinetic and thermodynamic parameters of physicochemical processes and deal with non-Arrhenius behavior.

## 6. Conclusions and Perspectives

This Special Issue’s multidisciplinary contributions highlight that the theory of fuzzy set and fuzzy logic are valuable conceptual tools to understand the molecular and supramolecular world. Of course, quantum-mechanics already exists for this purpose, but fuzzy logic is becoming an alternative approach that might have still undiscovered common points with quantum logic [37,38]. Fuzzy logic appears particularly suitable for dealing with conformers. Although this approach is in its infancy, it is worthwhile pursuing it. It will allow us to describe any cell’s activities, the constitutive elements of the human nervous system, and the immune system’s performances more deeply. Such knowledge will be translated into new strategies to control the cellular processes and develop chemical artificial intelligence and chemical robots [6]. If cutting-edge technologies emerge from this approach, then, biomolecular, supramolecular, and systems chemistry will surely be considered fuzzy worldwide!
